# Cytokine Profiles in Cervical Intraepithelial Neoplasia: Associations with Lesion Severity and Exploratory Treatment Response

**DOI:** 10.3390/diagnostics16203261

**Published:** 2026-10-09

**Authors:** Attila Louis Major, Aleksandra Riger, Nataliy Kharchenko, Antonova Irina, Vasilii Khammad, Natalia Riger, Nikolay Riger, Andrey Kaprin

**Affiliations:** 1 Femina Gynaecology Centre, 1205 Geneva, Switzerland; 2 Department of Obstetrics and Gynecology, Urgench Branch of Tashkent Medical Academy, Urgench 220100, Uzbekistan; 3 Department of Oncology and Interventional Radiology named after Academician V. P. Kharchenko, Institute of Medicine, Peoples’ Friendship University of Russia named after Patrice Lumumba (RUDN University), 117198 Moscow, Russia; aleksriger96@mail.ru (A.R.); nata2305@mail.ru (N.K.); mnioi@mail.ru (A.K.); 4 Russian Scientific Center of Roentgen Radiology (RSCRR), 117997 Moscow, Russia; iran24@yandex.ru; 5 James Cancer Hospital and Solove Research Institute, Columbus, OH 43210, USA; vas.khammad@gmail.com; 6 State Budgetary Healthcare Institution “Oncology Dispensary No. 2”, 354057 Sochi, Russia; riger_63@mail.ru; 7 Federal Research Center of Nutrition, Biotechnology and Food Safety, 109240 Moscow, Russia; rieger_63@mail.ru

**Keywords:** low-grade squamous intraepithelial lesion (LSIL), high-grade squamous intraepithelial lesion (HSIL), cervical intraepithelial neoplasia (CIN), high-risk human papillomavirus (hrHPV), minor colposcopic lesion, major colposcopic lesion, cytokines, exploratory biomarkers, IL-1β, IP-10, MCP-1, FGF

## Abstract

**Background:** Persistent infection with high-risk human papillomavirus (hrHPV) is the principal cause of cervical intraepithelial neoplasia (CIN). Cytokines regulate antiviral immunity and tissue remodeling, and cytokine profiling may provide additional information about the biology of cervical lesions. **Objective:** To characterize cytokine profiles in cervical mucus and serum across grades of cervical intraepithelial neoplasia. **Methods:** The study included 90 women aged 18–80 years: 30 HPV-negative women with negative cytology (Control) and 60 HPV-positive women with histologically confirmed low-grade (LSIL, *n* = 30; Gr-1) or high-grade (HSIL, *n* = 30; Gr-2) squamous intraepithelial lesions. Participants were also stratified by minor (grade 1) or major (grade 2) colposcopic findings. An exploratory subgroup of 15 women with HPV16/18-positive HSIL received antiviral and immunomodulatory therapy. Concentrations of 27 cytokines (pg/mL)—FGF, eotaxin, G-CSF, GM-CSF, IFN-γ, IL-1ra, IL-1β, IL-2, IL-4, IL-5, IL-6, IL-7, IL-8, IL-9, IL-10, IL-12(p70), IL-13, IL-15, IL-17A, IP-10, MCP-1, MIP-1α, MIP-1β, PDGF-BB, RANTES, TNF-α, and VEGF—were measured in serum and cervical mucus using the Bio-Plex Pro Human Cytokine 27-plex assay (Bio-Rad, USA) on a Luminex 200 platform. Statistical analyses were performed using SPSS version 23.0 and Microsoft Excel 365. Results are reported as medians and interquartile ranges [Q1–Q3]. A two-sided *p*-value < 0.05 was considered statistically significant. **Results:** Exploratory multivariate analyses identified IL-1β, IP-10, MCP-1, and FGF as the cytokines most strongly associated with lesion severity and colposcopic findings. In cervical mucus, HSIL was associated with the highest IL-1β concentration (0.83 pg/mL [0.18–4.91] vs. 0.30 pg/mL [0.04–0.69] in controls; *p* < 0.001) and the lowest IP-10 concentration (3.07 pg/mL [1.02–13.16] vs. 25.12 pg/mL [5.01–42.40]; *p* < 0.001). The highest mucus FGF concentration was observed in LSIL (1.73 pg/mL [1.33–2.27] vs. 0.32 pg/mL [0.17–0.59] in controls; *p* < 0.001), whereas MCP-1 was lower in LSIL than in HSIL (2.38 pg/mL [1.57–3.54] vs. 5.38 pg/mL [3.12–10.55]; *p* < 0.05). Similar patterns were observed in serum, except that IL-1β was lower than in controls. After treatment, HPV was undetectable in 10 women and remained detectable in 5. The overall cytokine profile changed only modestly. Among the five women with persistent HPV infection, cervical mucus MCP-1 was lower after treatment than before treatment (0.09 pg/mL [0.01–0.41] vs. 0.37 pg/mL [0.08–0.73]; *p* < 0.01). **Conclusions**: These findings show associations between lesion severity, colposcopic findings, and compartment-specific cytokine patterns. Because the study was cross-sectional and the treatment subgroup was small and uncontrolled, the results should be considered exploratory and require longitudinal validation.

## 1. Introduction

Chronic inflammation is increasingly recognized as a hallmark of cancer and contributes to tumor initiation, promotion, and progression [1,2]. Cytokines are small regulatory proteins that mediate intercellular communication and coordinate immune and inflammatory responses. Under physiological conditions, cytokine signaling is tightly regulated to produce an effective but self-limited immune response. Persistent dysregulation of cytokine networks, however, may sustain inflammatory signaling and create a microenvironment that favors cellular transformation and tumor development [3].

Cytokines can exert both tumor-suppressive and tumor-promoting effects. Proinflammatory cytokines, including interleukin (IL)-6, tumor necrosis factor (TNF)-α, and IL-1β, can enhance proliferation, inhibit apoptosis, promote angiogenesis, and contribute to tissue remodeling through pathways such as NF-κB and STAT3 [4,5]. Conversely, immunoregulatory and anti-inflammatory cytokines, particularly IL-10 and Th2-associated mediators, may facilitate immune evasion by suppressing effective antiviral and antitumor responses. Disrupted cytokine regulation may impair clearance of infected and dysplastic epithelial cells and thereby contribute to cervical epithelial transformation and lesion progression [6].

Cervical carcinogenesis is driven by persistent hrHPV infection and modified by inflammatory cofactors. More than 90% of cervical cancers are attributable to persistent hrHPV infection, particularly HPV16 and HPV18 [7]. Nevertheless, only a subset of infections persists and progresses to CIN or invasive carcinoma, indicating that additional host, environmental, and immunological factors contribute. These factors include genital infections such as Chlamydia trachomatis, multiple sexual partners, high parity, HIV-associated immunosuppression, and tobacco use [8,9]. Many may promote chronic mucosal inflammation and disrupt local immune control, thereby facilitating HPV persistence and lesion progression.

The local microenvironment of HPV-associated cervical lesions reflects interactions among infected epithelial cells, immune cells, and stromal components. Persistent hrHPV infection, viral genotype, viral burden, and local immune responses can alter epithelial signaling and the release of cytokines, chemokines, and growth factors while enabling sustained expression of viral oncoproteins. These mediators influence immune-cell recruitment, antiviral responses, angiogenesis, stromal remodeling, and epithelial-cell survival [10]. Such alterations may be present in both LSIL and HSIL, before the development of invasive cervical cancer [11].

Despite growing interest in cytokine-mediated mechanisms of HPV-associated carcinogenesis, the clinical implications of interactions among hrHPV genotypes, viral burden, and cytokine responses remain incompletely understood. Cytokine patterns associated with lesion severity may provide insight into the immunological microenvironment of cervical neoplasia, but their prognostic or diagnostic value requires prospective validation [12].

CIN develops in the setting of persistent infection with hrHPV genotypes such as HPV16, HPV18, HPV31, and HPV33. Viral oncoproteins E6 and E7 disrupt p53 and retinoblastoma protein (pRb) signaling, promoting genomic instability and abnormal cell-cycle control [13].

Cytokines implicated in cervical carcinogenesis include proinflammatory mediators such as TNF-α, IL-1β, IL-6, IL-17, and IL-8, as well as immunoregulatory mediators such as TGF-β and IL-10. IL-6 and IL-17 may promote angiogenesis, survival, and tissue remodeling. IL-17-producing T helper cells recruit neutrophils and myeloid cells, which can release proteases and reactive oxygen species that damage tissue architecture. Persistent hrHPV infection in lesions progressing to CIN2/3 has been associated with reinforced TNF-α, IL-1β, IL-6, IL-17, and IL-8 signaling, while TGF-β and IL-10 may suppress effective immune responses. IL-6 activates the JAK/STAT3 axis, which promotes proliferation, survival, epithelial–mesenchymal transition, and VEGF expression. In cancer cells and antigen-presenting cells, sustained STAT3 signaling can impair antigen presentation and antigen-specific T-cell responses, thereby contributing to immune tolerance [14].

Epithelial cells in the cervical transformation zone produce mediators such as IL-6, IL-8, and TGF-β that shape local immune-cell recruitment and function [15,16]. Langerhans cells and other antigen-presenting cells may be reduced or functionally altered, favoring an IL-10-biased response over protective IL-2/IFN-γ signaling. Macrophages and myeloid cells can combine proinflammatory matrix remodeling with immunosuppression and angiogenesis through TNF-α, IL-1β, IL-6, IL-10, and TGF-β [17,18]. Th2 and regulatory T-cell predominance, with contributions from IL-17, may facilitate persistence of CIN lesions [19,20]. These observations support investigation of cytokine profiles across lesion grades but do not, by themselves, establish progression to invasion.

## 2. Materials and Methods

### 2.1. Study Population

The study included 90 women aged 18–80 years who were consecutively enrolled in the Department of Oncology and Radiology at the Russian Scientific Center of Roentgen Radiology between 2024 and 2025. All participants provided written informed consent. The study protocol was approved by the local ethics committee, and the study was conducted in accordance with the Declaration of Helsinki.

Exclusion criteria included abnormal uterine bleeding (metrorrhagia), current use of combined oral contraceptives, pregnancy or lactation, autoimmune diseases or immunodeficiency disorders, clinical signs of cervical inflammation, sexually transmitted infections, use of immunosuppressive drugs within the previous 6 months, a history of malignancy or antitumor therapy, prior treatment for HPV infection, and prior surgical treatment for CIN. Participants were instructed to abstain from sexual intercourse for at least 24 h before sample collection and had not used antibiotics or topical vaginal or cervical agents during the month preceding sampling.

The control group (C) comprised 30 HPV-negative women with normal cervical cytology (negative for intraepithelial lesion or malignancy, NILM). The study groups comprised 60 HPV-positive women with histologically confirmed cervical intraepithelial lesions: 30 with LSIL/CIN1 (Gr-1) and 30 with HSIL/CIN2–3 (Gr-2). All 60 women in the LSIL and HSIL groups underwent histopathological examination. Group assignment was based on histologically confirmed CIN1 (LSIL) and CIN2–3 (HSIL), respectively.

All participants underwent cervical cytology, HPV testing, colposcopy, and immunocytochemical assessment of p16/Ki-67 co-expression.

Fifteen women with HPV16/18-positive, histologically confirmed HSIL received an exploratory conservative regimen used in the Russian Federation. During one month, these 15 participants received oral inosine pranobex 1000 mg (two tablets) three times daily for 10 days, with 10-day intervals between courses, for three courses in total, together with intravaginal 3,3′-diindolylmethane (DIM) suppositories at 100 mg twice daily [21,22]. After treatment, participants underwent repeat cervical cytology and HPV testing. This uncontrolled exploratory regimen was not evaluated as a replacement for guideline-directed excisional treatment.

### 2.2. Cytological Examination and Immunocytochemistry

Cervical specimens were collected using the CellPrep Pap Test system (Biodyne Co., Ltd., Seoul, Republic of Korea). Cytological evaluation followed the 2014 Bethesda System. Additional slides for immunocytochemistry were prepared from the residual preservative solution. Co-expression of p16 and Ki-67 was assessed using the CINtec^®^ PLUS Cytology Kit (Roche Diagnostics, Rotkreuz, Switzerland) on an automated BenchMark XT immunostainer (Ventana Medical Systems, Inc., Tucson, AZ, USA) according to the manufacturer’s instructions. A specimen was considered positive when at least one epithelial cell showed simultaneous p16 and Ki-67 expression.

### 2.3. HPV Detection and Genotyping

HPV genotyping was performed by real-time polymerase chain reaction using the Kvant-21 assay (DNA-Technology LLC, Moscow, Russia), which detects low- and high-risk HPV genotypes. High-risk HPV infection was additionally confirmed using the cobas^®^ HPV test (Roche Diagnostics, Rotkreuz, Switzerland), a multiplex real-time PCR assay that individually identifies HPV16 and HPV18 and concurrently detects 12 other hrHPV genotypes (31, 33, 35, 39, 45, 51, 52, 56, 58, 59, 66, and 68). β-globin served as an internal control of specimen adequacy.

### 2.4. Colposcopic and Histopathological Assessment

All participants underwent extended colposcopy according to standard protocols using 3–5% acetic acid and Lugol’s iodine (Schiller test). Colposcopic findings were classified according to the 2011 terminology of the International Federation for Cervical Pathology and Colposcopy (IFCPC).

Colposcopic severity was additionally assessed using the Swede score, which incorporates acetowhiteness, lesion size, lesion margins, vascular pattern, and iodine staining. Scores of 0–4 were classified as normal or minor changes (CIN1 or less), scores of 5–6 as major non-invasive changes (CIN2 or worse), and scores of 7–10 as major changes suspicious for invasion. Targeted cervical biopsy was performed when abnormal colposcopic findings were present, and histopathology was used as the reference standard for CIN diagnosis.

### 2.5. Cytokine Analysis

Serum and cervical mucus specimens were prepared according to the recommendations of Bio-Rad Laboratories, Inc. (Hercules, CA, USA). Cervical mucus was collected using sterile Pasteur pipettes into 0.6 mL tubes (Nest, Guangzhou, China). A 25 μL aliquot was added to 100 μL of the sample diluent supplied with the commercial assay and stored at −25 °C. Blood was collected into VACUETTE tubes (Greiner Bio-One, Vienna, Austria), centrifuged at 1000× *g* for 10 min at 25 °C, and 125 μL serum aliquots were stored at −25 °C.

Before multiplex analysis, serum and cervical mucus samples were centrifuged for 10 min at 10,000× *g* and 4 °C according to the manufacturer’s instructions; 25 μL aliquots were then analyzed. Cytokines (IFN-γ, TNF-α, IL-1ra, IL-1β, IL-2, IL-4, IL-5, IL-6, IL-7, IL-9, IL-10, IL-12[p70], IL-13, IL-15, and IL-17A), chemokines (eotaxin, IL-8, IP-10, MCP-1, MIP-1α, MIP-1β, and RANTES), and growth factors (FGF, PDGF-BB, VEGF, G-CSF, and GM-CSF) were quantified in cervical mucus and serum (pg/mL) using the Bio-Plex Pro Human Cytokine 27-plex Assay (Bio-Rad Laboratories, Inc., Hercules, CA, USA) on a Luminex 200 analyzer (Luminex Corporation, Austin, TX, USA) with xMAP technology.

### 2.6. Statistical Analysis

Statistical analyses were performed using SPSS version 23.0 (IBM Corp., Armonk, NY, USA) and Microsoft Excel 365.

The primary between-participant analyses considered three-level factors: lesion group (control, LSIL/CIN1, and HSIL/CIN2–3), HPV group (HPV-negative control, HPV16/18, and non-16/18 hrHPV genotypes 31–68). Cytokine concentrations in serum and cervical mucus were the dependent variables. An additional paired before-and-after analysis evaluated within-participant changes in women with HSIL according to post-treatment HPV status. For each participant, the difference between post-treatment and pretreatment cytokine concentrations was calculated and used as the dependent variable. The treatment-outcome analysis was exploratory and unbalanced.

Data distributions were assessed using the Shapiro–Wilk test, and homogeneity of variances was assessed using Levene’s test. Continuous variables are reported as medians and interquartile ranges [Q1–Q3] because several cytokine measures were not normally distributed.

Three independent groups were compared using one-way analysis of variance when assumptions of normality and homogeneity of variance were met; otherwise, the Kruskal–Wallis test was used. Effect sizes were summarized using η^2^ and ω^2^. Linear and quadratic contrasts were used to characterize trends across ordered groups. Cytokines identified during screening were evaluated jointly using Hotelling’s T^2^, followed by linear discriminant analysis (LDA) of z-standardized variables to identify linear combinations that best separated the groups. Where required to reduce sensitivity to non-normality and heteroscedasticity, quantitative outcomes were rank-transformed using aligned rank transformation. Pairwise parametric comparisons used Welch’s two-sample t-test with Bonferroni correction; nonparametric comparisons used Dunn’s post hoc test with Bonferroni correction. A two-sided *p*-value < 0.05 was considered statistically significant. These analyses were exploratory and did not adjust for the clinical and behavioral covariates listed in Table 1.

## 3. Results

First, we compared cervical mucus cytokine profiles among women with normal cervical epithelium, LSIL, and HSIL. Exploratory multivariate analysis identified IL-1β, IP-10, FGF, and MCP-1 as the cytokines most strongly associated with lesion severity and colposcopic findings (η^2^ ≥ 0.14; *p*-values Kruskal–Wallis < 0.05; Figure 1 and Figure 2 and Table 2). Joint analysis of these four cytokines showed a significant response to lesion severity by Hotelling’s T^2^ (*p* = 0.0004). LDA of the standardized four-cytokine profile identified the variables contributing most strongly to separation of the lesion-severity groups.

Women with HSIL had a higher cervical mucus IL-1β concentration than controls (0.83 pg/mL [0.18–4.91] vs. 0.30 pg/mL [0.04–0.69]; *p* < 0.001; Figure 1 and Table 2). Conversely, cervical mucus IP-10 was lower in HSIL than in controls (3.07 pg/mL [1.02–13.16] vs. 25.12 pg/mL [5.01–42.40]; *p* < 0.001; Figure 2 and Table 2).

Across the four selected cytokines (Table 2), histologically confirmed LSIL was associated with higher IL-1β (*p* < 0.05) and FGF (*p* < 0.001) concentrations and lower IP-10 (*p* < 0.05) and MCP-1 (*p* = 0.08) concentrations than controls. Similar directional changes were observed in HSIL, although the MCP-1 difference from controls was not statistically significant despite a higher median. FGF was also higher in HSIL than in controls, but the highest concentration was observed in LSIL (*p* < 0.05 for LSIL vs. HSIL; Table 2).

When participants were stratified by colposcopic severity using the Swede score, women with minor grade 1 findings (0–4 points) had the highest cervical mucus FGF concentration (1.73 pg/mL [1.33–2.27]), compared with women with major grade 2 findings (≥5 points; 0.90 pg/mL [0.73–1.63]; *p* < 0.05) and controls (0.32 pg/mL [0.17–0.59]; *p* < 0.001; Figure 3 and Table 2). In the LDA, variation in cervical mucus FGF contributed to separation of the lesion-severity groups and may represent a candidate feature associated with SIL severity.

Other cytokine differences were also observed across colposcopic-severity groups (Figure 3 and Table 2). Cervical mucus IL-1β increased from 0.60 pg/mL [0.23–0.88] in grade 1 lesions to 0.83 pg/mL [0.18–4.91] in grade 2 lesions (*p* < 0.05). IP-10 was lower in both minor (5.14 pg/mL [1.53–7.74]; *p* < 0.05) and major (3.07 pg/mL [1.02–13.16]; *p* < 0.001) lesions than in controls, but did not differ significantly between the two lesion groups. MCP-1 tended to be lower in minor lesions than in controls (2.38 pg/mL [1.57–3.54] vs. 3.96 pg/mL [2.69–6.47]; *p* = 0.08) and was higher in major than in minor lesions (*p* < 0.05). Neither lesion group differed significantly from controls for MCP-1.

Serum patterns of IL-1β, IP-10, FGF, and MCP-1 differed in several respects from those observed in cervical mucus (Figure 4 and Table 3). Serum FGF was higher in women with major colposcopic findings than in controls and women with minor findings (both *p* < 0.05). Serum IP-10 and MCP-1 were lower in women with minor findings than in controls and women with major findings (all *p* < 0.05; Figure 4; Table 3). In contrast to the pattern in cervical mucus, serum IL-1β was lower in both lesion groups than in controls, with the largest difference observed for minor lesions (0.04 pg/mL [0.03–0.06] vs. 0.10 pg/mL [0.08–0.13]; *p* < 0.05). Serum IL-1β did not differ significantly between minor and major lesions (Figure 4; Table 3).

Fifteen women with HPV16/18-positive HSIL received the exploratory antiviral and immunomodulatory regimen. HPV was undetectable after treatment in 10 women and remained detectable in 5. Overall, cervical mucus cytokine profiles changed only modestly. Among the five women with persistent HPV infection, MCP-1 decreased from 0.37 pg/mL [0.08–0.73] before treatment to 0.09 pg/mL [0.01–0.41] after treatment (*p* < 0.01; Figure 5). Because this subgroup was small and lacked an untreated comparator, the observed change cannot be attributed to the regimen and may reflect within-person variability or the natural history of HPV infection. In the LDA, the largest standardized coefficients were positive for FGF (0.015) and negative for MCP-1 (−0.015), a pattern consistent with lower FGF and higher MCP-1 in more severe lesions. This finding is exploratory. A further study should analyze if it is confirmed that MCP-1 can be useful to test in blood.

## 4. Discussion

Cytokines have pleiotropic and context-dependent functions, and the same mediator may exert different effects across stages of cervical neoplasia. Accordingly, altered cytokine concentrations and combinations may reflect changes in the local immune and stromal environment, but cross-sectional measurements cannot establish oncogenic progression or prognosis [23].

In this study, IL-1β, IP-10, MCP-1, and FGF emerged as candidate cytokines associated with lesion severity. These mediators are produced by monocytes/macrophages, lymphocytes, endothelial cells, fibroblasts, epithelial cells, and other cell populations involved in local and systemic immune responses and tissue remodeling [24,25,26].

The higher cervical mucus IL-1β concentration may reflect increased production by activated monocytes/macrophages, fibroblasts, or epithelial cells and may involve NLRP3 inflammasome signaling during persistent HPV infection [27]. This mechanistic interpretation remains inferential because cellular sources and pathway activation were not measured directly.

In the cervical microenvironment, NLRP3 signaling can have context-dependent effects, and hrHPV may alter this pathway during immune evasion and malignant transformation. The tumor immune microenvironment is initiating a dual effect of some cytokines and proteins. This dual behavior may change the response to immunotherapy in ovarian, endometrial, and cervical cancer [28,29,30]. IP-10 (CXCL10) is produced by macrophages and activated epithelial cells in response to interferons. MCP-1 (CCL2) is produced by macrophages and epithelial cells and recruits monocytes and other immune cells. FGF is released by several stromal and epithelial cell types and participates in tissue repair and angiogenesis [31].

The higher local IL-1β concentration observed in cervical mucus (Figure 1 and Figure 3) may also reflect recruitment or activation of macrophages and T cells. IL-1β and TNF-α can activate p38 MAPK/NF-κB signaling, which regulates additional proinflammatory cytokines, chemokines, and growth factors. NLRP3-mediated IL-1β production may therefore participate in a positive-feedback inflammatory circuit [32]. These pathways were not measured directly in the present study.

Lower IP-10 in HSIL and in both minor and major colposcopic lesions, together with compartment-specific IL-1β patterns in cervical mucus and serum (Figure 1, Figure 2, Figure 3 and Figure 4), may reflect altered local antiviral signaling in CIN. These associations do not establish a causal role in lesion development.

IP-10 promotes leukocyte trafficking and supports interferon-associated antiviral and antitumor responses [33]. Reduced IP-10 may therefore be compatible with weaker IFN-γ-associated signaling, although IFN-γ pathway activity was not directly assessed. Growth factors such as VEGF, FGF, and PDGF-BB may contribute to angiogenesis and tissue remodeling. In our data, cervical mucus FGF was highest in women with minor lesions and remained higher in HSIL than in controls (Figure 3 and Table 2). The combination of lower IP-10 and altered FGF is therefore hypothesis-generating and requires mechanistic and longitudinal validation.

Cytokine networks can exert complementary or opposing effects on antitumor surveillance. In chronic HPV infection, viral oncoproteins and microenvironmental signals may promote M2-like macrophage polarization [34]. Such macrophages can release IL-10 and other immunosuppressive mediators that favor immune escape and viral persistence [35].

MCP-1 was lower in minor colposcopic lesions than in major lesions and showed a nonsignificant reduction relative to controls. MCP-1 was also lower after treatment in the five women who remained HPV-positive. Because the treatment subgroup was small and uncontrolled, this before-and-after observation should not be interpreted as evidence of a therapeutic effect. Biologically, MCP-1 is a potent chemoattractant for monocytes, macrophages, memory T cells, and dendritic cells and can activate p38 MAPK, ERK1/2, and JNK signaling. Higher MCP-1 in more severe lesions may therefore reflect increased inflammatory-cell recruitment, but the present data do not identify the cellular source.

The observed differences in IL-1β, IP-10, MCP-1, and FGF are compatible with interrelated inflammatory, antiviral, and tissue-remodeling pathways implicated in HPV-associated carcinogenesis. However, this study included no invasive cancers, no longitudinal assessment of progression or regression, and no diagnostic-accuracy analysis. The findings therefore support associations with current lesion severity rather than transformation, prognosis, or individualized treatment selection.

The opposite direction of IL-1β in cervical mucus and serum may indicate compartment-specific immune regulation. A localized mucosal inflammatory response can coexist with lower circulating concentrations because tissue production, cellular uptake, diffusion, and systemic clearance differ across compartments. The present study did not measure these mechanisms, so the pattern should be interpreted as hypothesis-generating rather than evidence of immune isolation.

This study has several limitations. Its cross-sectional design precludes conclusions about progression or regression. Associations of smoking, age at first intercourse, parity, infections, and number of sexual partners with cytokine concentrations were not evaluated because this exploratory study was not designed or powered for multivariable analysis. The absence of an adjusted analysis has now been acknowledged as a limitation. Therefore, residual confounding cannot be excluded and should be considered when interpreting the findings. Sample collection was not standardized for menstrual-cycle phase or douching. The 27-cytokine panel generated multiple comparisons, and the small uncontrolled treatment subgroup was susceptible to spontaneous HPV clearance and regression to the mean. No participants with invasive cervical cancer were included, and no longitudinal diagnostic or prognostic performance measures were estimated.

## 5. Conclusions

Cervical mucus concentrations of IL-1β, IP-10, MCP-1, and FGF differed across lesion-severity groups, and several serum patterns were directionally distinct from those in cervical mucus. These cytokines should be regarded as exploratory biomarkers associated with current lesion severity. Their diagnostic, prognostic, and treatment-predictive value has not been established. The small uncontrolled treatment subgroup permits only descriptive before-and-after observations and does not support replacing standard excisional management of HSIL. Prospective longitudinal studies with clinically comparable groups, adjustment for relevant confounders, prespecified multiplicity control, diagnostic-accuracy analyses, and independent validation are required before these cytokines can be used for risk stratification or treatment-response monitoring.

## Figures and Tables

**Figure 1:**
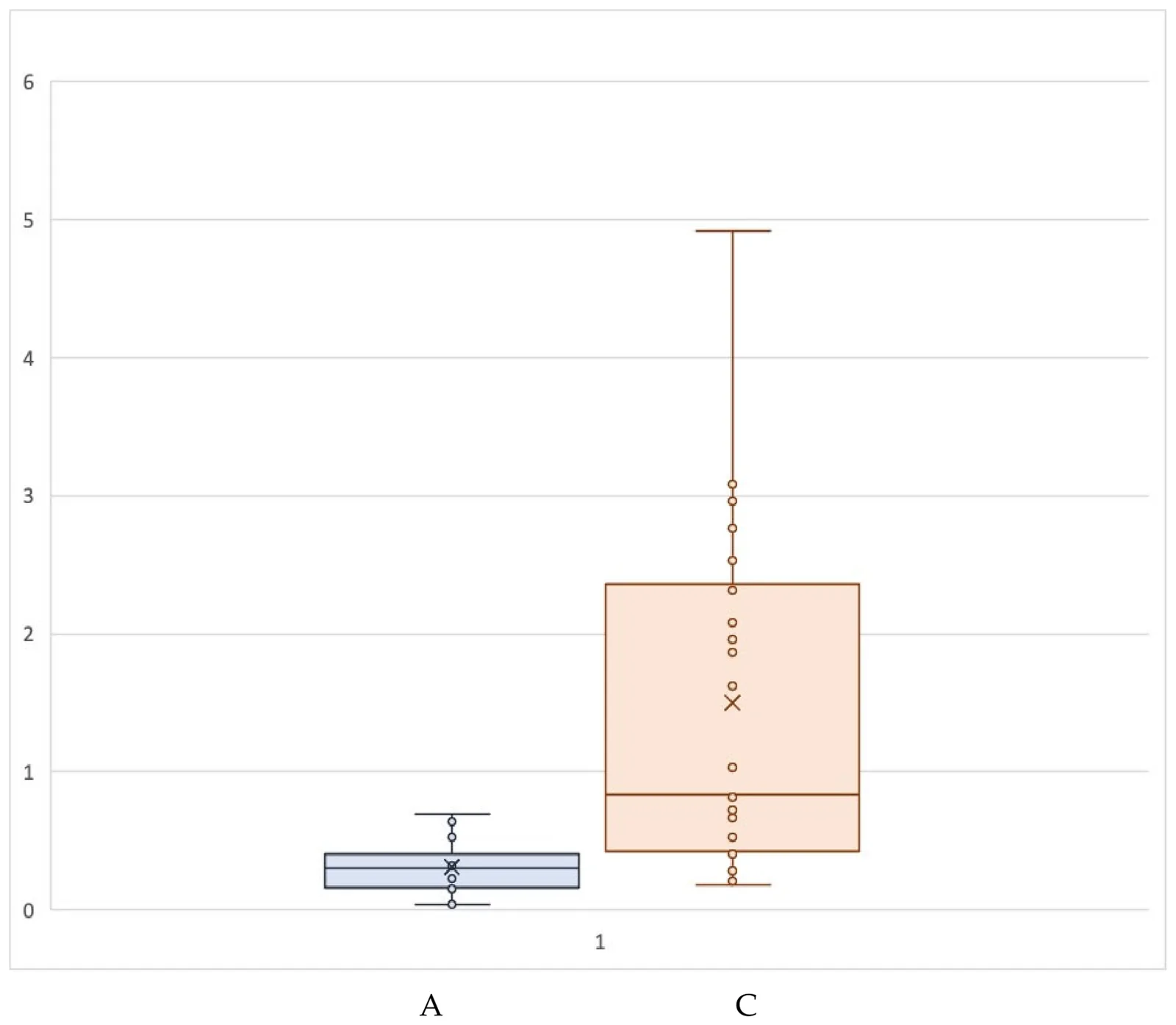
Cervical mucus IL-1β concentration in women with HSIL/CIN2–3 and controls (NILM). The *y*-axis shows IL-1β concentration (pg/mL); A, control group; C, HSIL/CIN2–3 group. The difference between groups A and C was statistically significant (*p* < 0.001). Boxes represent the median and interquartile range (Q1–Q3), and whiskers indicate the minimum and maximum values.

**Figure 2:**
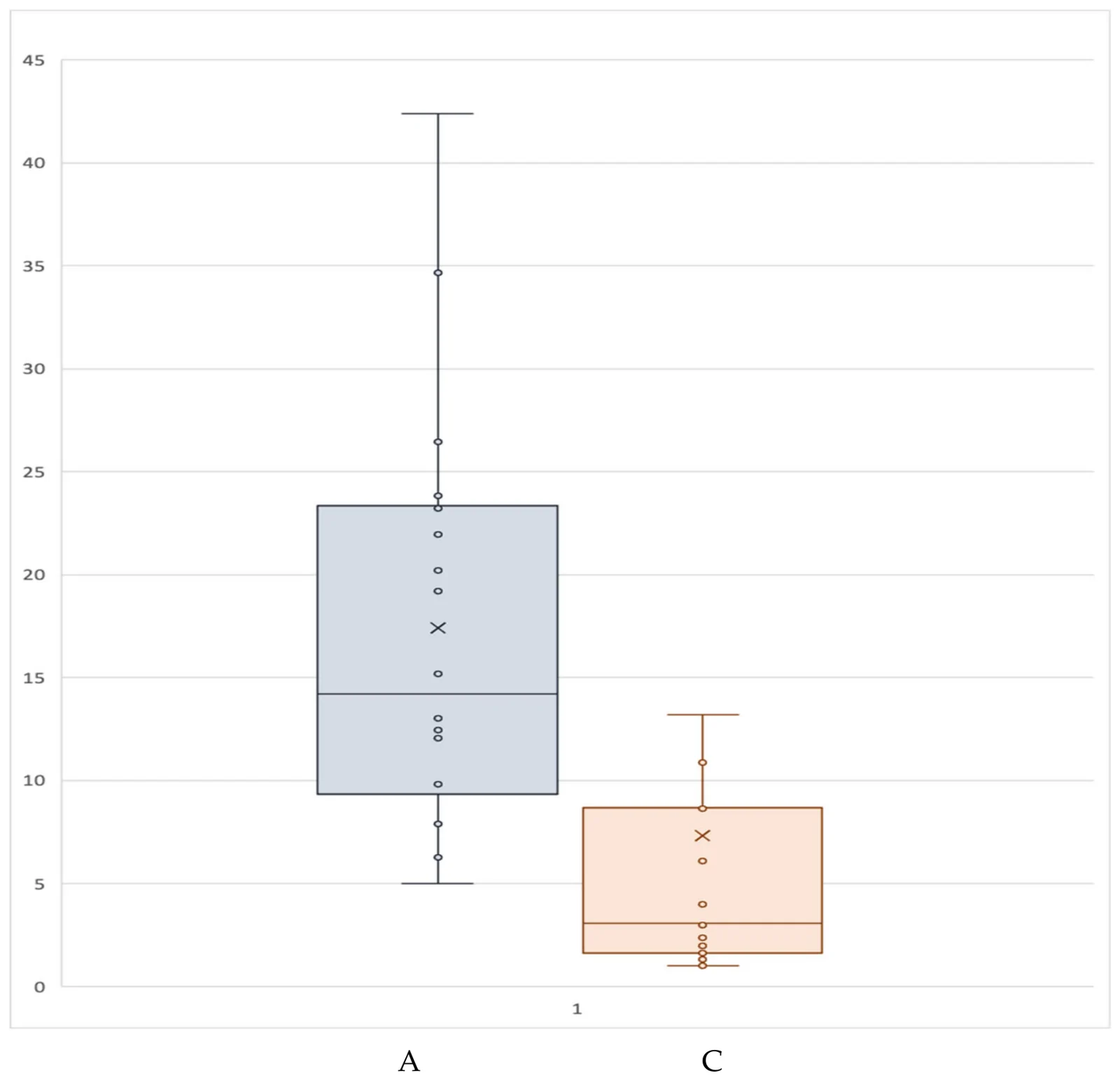
Cervical mucus IP-10 concentration in women with HSIL/CIN2–3 and controls (NILM). The *y*-axis shows IP-10 concentration (pg/mL); A, control group; C, HSIL/CIN2–3 group. The difference between groups A and C was statistically significant (*p* < 0.001). Boxes represent the median and interquartile range (Q1–Q3), and whiskers indicate the minimum and maximum values.

**Figure 3:**
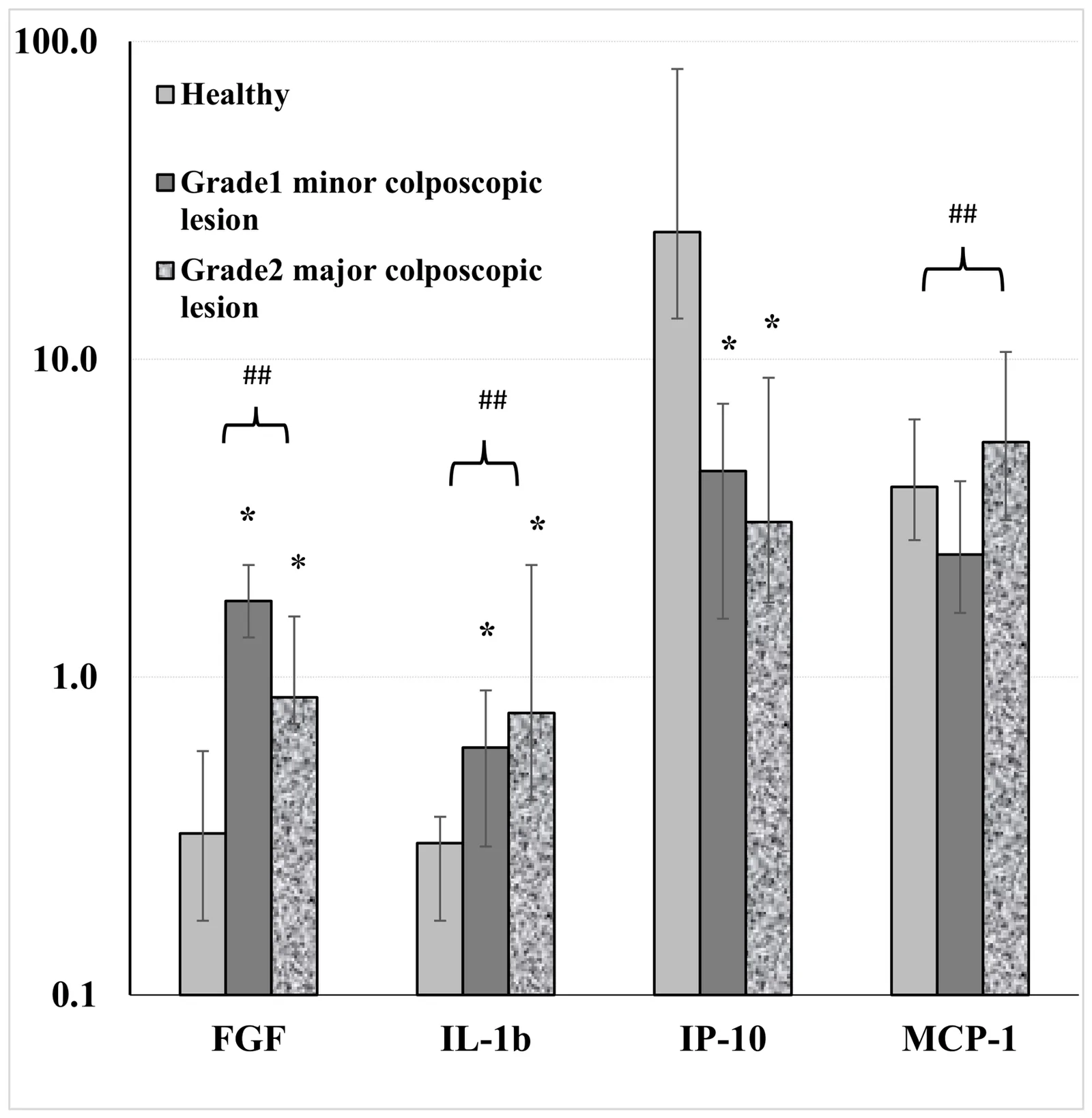
Cervical mucus concentrations of FGF, IL-1β, IP-10, and MCP-1 according to colposcopic severity. * *p* < 0.05 for controls versus participants with colposcopic lesions; ## *p* < 0.05 for minor versus major colposcopic lesions.

**Figure 4:**
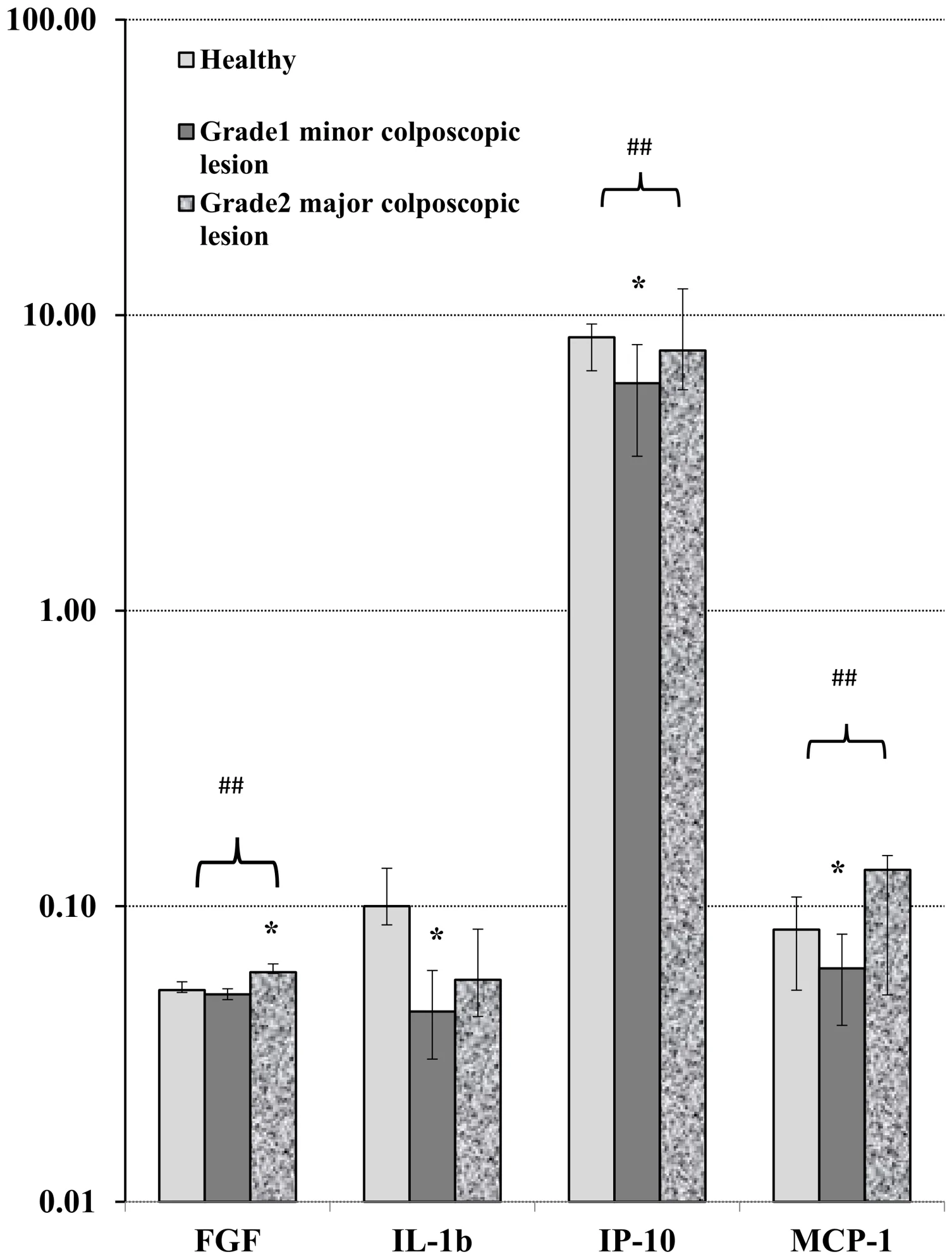
Serum concentrations of FGF, IL-1β, IP-10, and MCP-1 according to colposcopic severity. * *p* < 0.05 for controls versus participants with colposcopic lesions; ## *p* < 0.05 for minor versus major colposcopic lesions.

**Figure 5:**
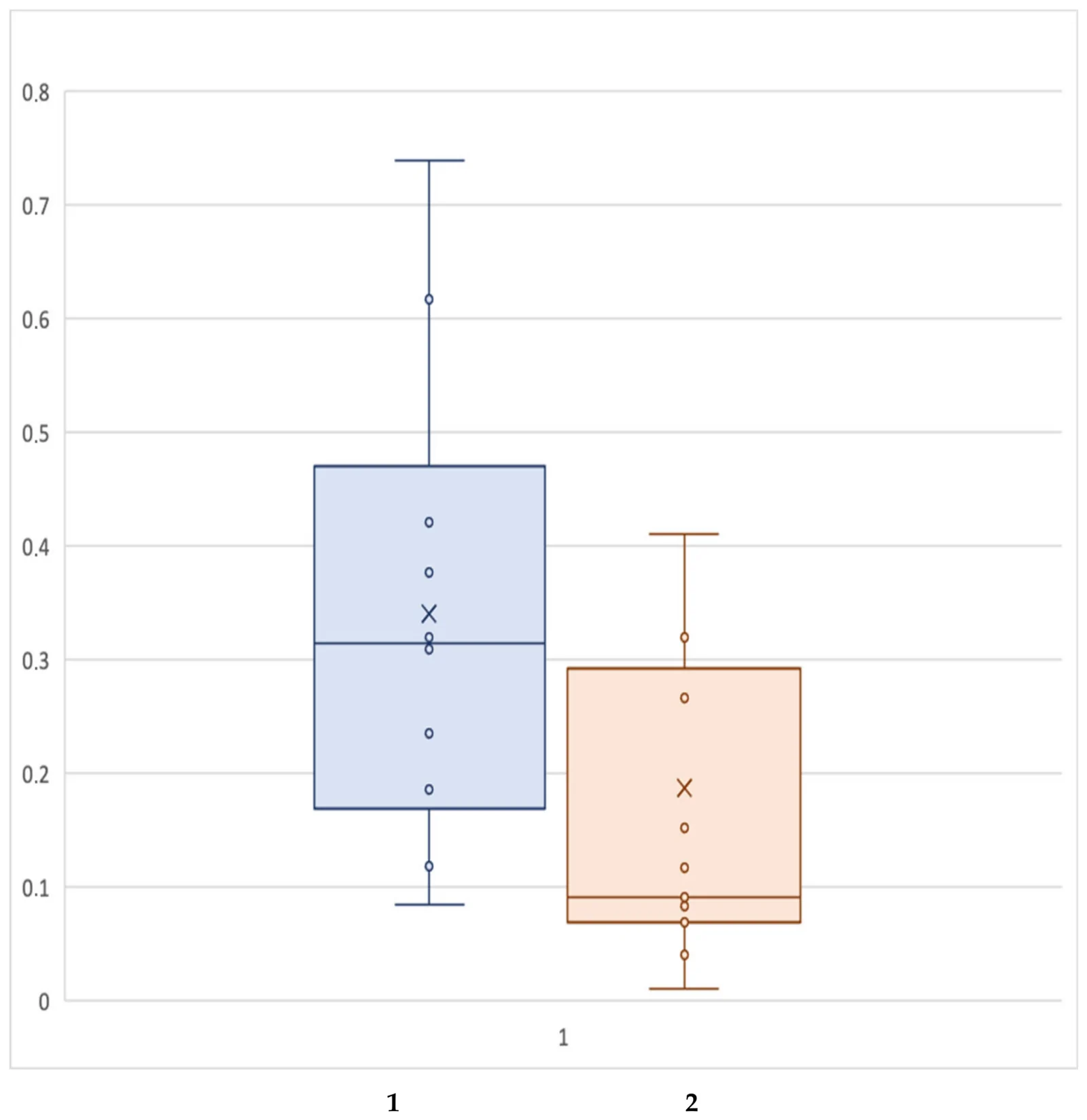
Cervical mucus MCP-1 concentration in the five women with persistent HPV16/18 infection: 1, before treatment; 2, after treatment.Boxes represent the median and interquartile range (Q1–Q3), and whiskers indicate the minimum and maximum values.

**Table 1:** Baseline patients’ characteristics.

	NILM (Control) (*n* = 30)	Group 1 (*n* = 30)	Group 2 (*n* = 30)	*p*. ANOVA, X^2^	*p*, Post hoc
Age, mean ± SD	30.3 ± 1.7	31.0 ± 1.0	31.7 ± 0.4	<0.001 *	C-1: 0.057 C-2: <0.001 1–2: 0.057
Smoking, *n* (%)	5 (16.7)	15 (50.0)	20 (66.7)	<0.001 ^^^	C-1: 0.017 C-2: <0.001 1–2: 0.342
Age of first sexual intercourse, mean ± SD	19.0 ± 0.8	17.0 ± 1.5	16.3 ± 1.9	<0.001 *	C-1: <0.001 C-2: <0.001 1–2: 0.16
Parity > 2, *n* (%)	3 (10.0)	7 (23.3)	9 (30.0)	0.154	
Infections, *n* (%)	9 (30.0)	13 (43.3)	15 (50.0)	0.277	
Multiple partners >1, *n* (%)	5 (16.7)	26 (86.7)	29 (96.7)	<0.001 ^^^	C-1: <0.001 C-2: <0.001 1–2: 0.438
HPV 16/18, *n* (%)	-	15 (50.0)	15 (50.0)	<0.001 ^^^	C-1: <0.001 C-2: <0.001 1–2: 1.0
- alone	-	12 (40.0)	11 (36.7)	<0.001 ^^^	C-1: <0.001 C-2: 0.002 1–2: 0.801
- with Low risk HPV (6, 11, 44)	-	3 (10.0)	4 (13.3)	0.12	C-1: 0.318 C-2: 0.134 1–2: 0.879
Other hrHPV, *n* (%)	-	15 (50.0)	15 (50.0)	<0.001 ^^^	C-1: <0.001 C-2: <0.001 1–2: 1.0
Cytology, *n* (%)					
- NILM	30 (100)	1 (3.3)	2 (6.7)	<0.001 ^^^	C-1: <0.001 C-2: <0.001 1–2: 0.755
- ASC-US	-	4 (13.3)	5 (16.7)	0.076	C-1: 0.195 C-2: 0.08 1–2: 0.901
- LSIL	-	9 (30.0)	9 (30.0)	0.003 ^^^	C-1: 0.008 C-2: 0.008 1–2: 1.0
- ASC-H	-	7 (23.3)	1 (3.3)	0.002 ^^^	C-1: 0.003 C-2: 0.882 1–2: 0.014
- HSIL	-	9 (30.0)	13 (43.3)	<0.001 ^^^	C-1: 0.012 C-2: <0.001 1–2: 0.397
Histology, *n* (%)					
- CIN 1	-	30 (100)	-	<0.001 ^^^	C-1: <0.001 C-2: 1.0 1–2: <0.001
- CIN 2–3	-	-	30 (100)	<0.001 ^^^	C-1: 1.0 C-2: <0.001 1–2: <0.001
Colposcopic lesion, *n* (%)					
- 0–4	30 (100)	30 (100)	4 (13.3)	<0.001 ^^^	C-1: 1.0 C-2: <0.001 1–2: <0.001
- 5–6	-	-	25 (83.3)	<0.001 ^^^	C-1: 1.0 C-2: <0.001 1–2: <0.001
- 7–10	-	-	1 (3.3)	0.372	C-1: 1.0 C-2: 0.442 1–2: 0.442
ICH (p16/ki67), *n* (%)					
- positive	-	9 (30.0)	16 (53.3)	<0.001 ^^^	C-1: 0.012 C-2: <0.001 1–2: 0.065
- negative	30 (100)	21 (70.0)	14 (46.7)		

Notes: *—statistically significant difference according to ANOVA, ^—statistically significant difference according to chi-square test.

**Table 2:** Concentrations of IL-1β, IP-10, FGF, and MCP-1 in cervical mucus.

	NILM (Control) (*n* = 30)	Group 1 (*n* = 30)	Group 2 (*n* = 30)	*p*. Kruskal–Wallis	*p*, Post hoc
	Me [Q_1_; Q_3_]	Me [Q_1_; Q_3_]	Me [Q_1_; Q_3_]		
IL-1β, pg/mL	0.30 [0.04; 0.69]	0.60 [0.23; 0.88]	0.83 [0.18; 4.91]	<0.001 ^#^	C-1: <0.05 C-2: <0.001 1–2: <0.05
IP-10, pg/mL	25.1 [5.01; 42.40]	5.1 [1.53–7.74]	3.2 [1.02; 13.16]	0.001 ^#^	C-1: <0.05 C-2: < 0.001 1–2: >0.1
FGF, pg/mL	0.32 [0.17; 0.59]	1.73 [1.33; 2.27]	0.90 [0.73; 1.63]	<0.001 ^#^	C-1: <0.001 C-2: <0.05 1–2: <0.05
MCP-1, pg/mL	4.0 [2.6; 6.7]	2.4 [1.57; 3.54]	5.4 [3.12; 0.55]	0.043 ^#^	C-1: 0.08 C-2: >0.1 1–2: <0.05

Notes: Concentrations are reported in pg/mL as medians [Q1–Q3]. IL-1β, interleukin-1β; IP-10, interferon-γ-induced protein 10; FGF, fibroblast growth factor; MCP-1, monocyte chemoattractant protein 1. ^#^—statistically significant difference according to Kruskal–Wallis’s test; The tabulated *p*-values compare each lesion group with the control group (C); LSIL (1) versus HSIL (2).

**Table 3:** Serum concentrations of IL-1β, IP-10, FGF, and MCP-1.

	NILM (Control) (*n* = 30)	Group 1 (*n* = 30)	Group 2 (*n* = 30)	*p*. Kruskal–Wallis	*p*, Post hoc
	Me [Q_1_; Q_3_]	Me [Q_1_; Q_3_]	Me [Q_1_; Q_3_]		
IL-1β, pg/mL	0.10 [0.08; 0.13]	0.04 [0.03; 0.06]	0.06 [0.04; 0.08]	0.016 ^#^	C-1: <0.05 C-2: >0.1 1–2: >0.1
IP-10, pg/mL	8.4 [6.49; 9.33]	5.88 [3.33; 7.95]	7.6 [5.4; 13.9]	0.015 ^#^	C-1: <0.05 C-2: >0.1 1–2: <0.05
FGF, pg/mL	0.052 [0.049; 0.054]	0.050 [0.048; 0.053]	0.060 [0.059; 0.064]	<0.001 ^#^	C-1: >0.1 C-2: <0.05 1–2: <0.05
MCP-1, pg/mL	0.08 [0.05; 0.11]	0.06 [0.04; 0.08]	0.13 [0.05; 0.15]	<0.001 ^#^	C-1: <0.05 C-2: 0.07 1–2: <0.05

Notes: Concentrations are reported in pg/mL as medians [Q1–Q3]. IL-1β, interleukin-1β; IP-10, interferon-γ-induced protein 10; FGF, fibroblast growth factor; MCP-1, monocyte chemoattractant protein 1. ^#^—statistically significant difference according to Kruskal–Wallis’s test; The tabulated *p*-values compare each lesion group with the control group (C); LSIL (1) versus HSIL (2).

## Data Availability

The original contributions presented in this study are included in the article. Further inquiries can be directed to the corresponding author.
